# Exploring the Neural Structures Underlying the Procedural Memory Network as Predictors of Language Ability in Children and Adolescents With Autism Spectrum Disorder and Attention Deficit Hyperactivity Disorder

**DOI:** 10.3389/fnhum.2020.587019

**Published:** 2020-12-10

**Authors:** Teenu Sanjeevan, Christopher Hammill, Jessica Brian, Jennifer Crosbie, Russell Schachar, Elizabeth Kelley, Xudong Liu, Robert Nicolson, Alana Iaboni, Susan Day Fragiadakis, Leanne Ristic, Jason P. Lerch, Evdokia Anagnostou

**Affiliations:** ^1^Holland Bloorview Kids Rehabilitation Hospital, Toronto, ON, Canada; ^2^Mouse Imaging Centre, The Hospital for Sick Children, Toronto, ON, Canada; ^3^Department of Paediatrics, Medical Sciences Building, University of Toronto, Toronto, ON, Canada; ^4^Psychiatry Research, The Hospital for Sick Children, Toronto, ON, Canada; ^5^Department of Psychiatry, University of Toronto, Toronto, ON, Canada; ^6^Department of Psychology, Queen’s University, Kingston, ON, Canada; ^7^Department of Psychiatry, Queen’s University, Kingston, ON, Canada; ^8^Department of Psychiatry, Schulich School of Medicine & Dentistry, University of Western Ontario, London, ON, Canada; ^9^Department of Medical Biophysics, University of Toronto, Toronto, ON, Canada

**Keywords:** ASD (autism spectrum disorder), ADHD (attention deficit hyperactivity disorder), structural language abilities, brain structure, procedural deficit hypothesis (PDH), structural MRI

## Abstract

**Introduction**: There is significant overlap in the type of structural language impairments exhibited by children with autism spectrum disorder (ASD) and children with attention deficit hyperactivity disorder (ADHD). This similarity suggests that the cognitive impairment(s) contributing to the structural language deficits in ASD and ADHD may be shared. Previous studies have speculated that procedural memory deficits may be the shared cognitive impairment. The procedural deficit hypothesis (PDH) argues that language deficits can be explained by differences in the neural structures underlying the procedural memory network. This hypothesis is based on the premise that the neural structures comprising the procedural network support language learning. In this study, we aimed to test the PDH in children with ASD, ADHD, and typical development (TD).

**Methods**: One hundred and sixty-three participants (ages 10–21): 91 with ASD, 26 with ADHD, and 46 with TD, completed standardized measures of cognitive and language ability as well as structural magnetic resonance imaging. We compared the structural language abilities, the neural structures underlying the procedural memory network, and the relationship between structural language and neural structure across diagnostic groups.

**Results**: Our analyses revealed that while the structural language abilities differed across ASD, ADHD, and TD groups, the thickness, area, and volume of the structures supporting the procedural memory network were not significantly different between diagnostic groups. Also, several neural structures were associated with structural language abilities across diagnostic groups. Only two of these structures, the inferior frontal gyrus, and the left superior parietal gyrus, are known to be linked to the procedural memory network.

**Conclusions**: The inferior frontal gyrus and the left superior parietal gyrus, have well-established roles in language learning independent of their role as part of the procedural memory system. Other structures such as the caudate and cerebellum, with critical roles in the procedural memory network, were not associated with structural language abilities across diagnostic groups. It is unclear whether the procedural memory network plays a fundamental role in language learning in ASD, ADHD, and TD.

## Introduction

It is estimated that 1 in every 66 children in Canada is diagnosed with autism spectrum disorder (ASD; Ofner et al., 2018). ASD is characterized by deficits in social interactions and social communication and restricted interests and/or repetitive behaviors (American Psychiatric Association, 2013). There is, however, a large subset of children with ASD who also exhibit language difficulties, not only concerning pragmatic abilities but with structural language (grammar) as well (Bishop and Norbury, 2002; Tager-Flusberg, 2006; Perovic et al., 2013). While the pragmatic impairments have been linked to deficits in theory of mind and its corresponding neural networks (Baron-Cohen, 2000), the neurocognitive underpinnings of the structural language deficits in ASD remain unknown.

Similar to ASD, co-occurring structural language deficits have also been reported in Attention Deficit Hyperactivity Disorder (ADHD; Kim and Kaiser, 2000; Wassenberg et al., 2010; Papaeliou et al., 2015). ADHD is one of the most commonly diagnosed neurodevelopmental disorders in children (Polancyzk et al., 2007; Redmond, 2016) and is characterized by impulsivity, inattention, and hyperactivity that negatively impact daily living (American Psychiatric Association, 2013). ADHD is often diagnosed with other comorbid disorders (Brown, 2000). Among the comorbidities reported in ADHD, language impairment is one of the most commonly reported co-occurring conditions (Mueller and Tomblin, 2012; Sciberras et al., 2014).

Given that poor language abilities in children with ASD and ADHD have been linked to poor social and academic outcomes in adolescence and adulthood (Guerts and Embrechts, 2008), identifying the mechanisms contributing to the structural language impairment in these disorders is critical.

### Structural Language Profiles of ASD and ADHD

Studies assessing the structural language abilities of children with ASD have reported deficits in the areas of morphology, syntax, and phonological processing (Tager-Flusberg, 2006). Morphosyntactic studies in ASD have shown that children with ASD often omit verb-related morphemes such as those specifying past tense (-ed) and third-person singular (-s) in spontaneous speech resulting in poorly formed sentences (Roberts et al., 2004; Tager-Flusberg, 2006). Difficulties processing complex sentence structures have also been reported in ASD. This deficit has been attributed to issues with interpreting long-distance dependencies including relative clauses, passives and, non-canonical word order (Whitehouse et al., 2008; Riches et al., 2010; Durrleman et al., 2015). Studies examining phonological processing suggest that children with ASD are more likely to have problems with detection, discrimination, and reproduction of speech sounds as evidenced by cluster reductions (e.g., ***sp***ider pronounced as ***p***ider), final consonant deletions (e.g., bea***t*** pronounced as be_) and gliding (e.g., ***l***eg pronounced as ***y***eg) on standardized measures of articulation as well as production errors on nonword repetition tasks (Kjelgaard and Tager-Flusberg, 2001; Tager-Flusberg, 2006; Whitehouse et al., 2008; Cleland et al., 2010).

Structural language deficits have also been reported in children with ADHD (Mueller and Tomblin, 2012; Sciberras et al., 2014). Children with ADHD have been reported to show poor sentence formulation and word structuring as a result of morphological weaknesses such as incorrect omission or insertion of function words and the improper specification of morphemes for verbs and nouns (Oram et al., 1999; Cohen et al., 2000). Syntactic issues have also contributed to their weak comprehension and construction of grammatical structures including word order violations and poor recall and interpretation of passive and relative clauses (Oram et al., 1999; Cohen et al., 2000; Kim and Kaiser, 2000). Phonological processing also appears to be commonly affected in children with ADHD as indicated by missing segments in their repetition of words and poor articulation of sounds (Oram et al., 1999; Cohen et al., 2000; Kim and Kaiser, 2000). Reading disabilities have also been reported in children with ADHD, which have been attributed to poor phonological awareness (Purvis and Tannock, 2000). There appears to be considerable overlap in the structural language impairments observed across ASD and ADHD.

### Procedural Deficit Hypothesis (PDH)

Given the parallels between the structural language deficits observed in ASD and ADHD, it could be hypothesized that the neurocognitive mechanism(s) underlying the structural language impairments are similar across the two disorders. Here, we speculate, as others have before (Ullman, 2004; Walenski et al., 2006; Nicolson and Fawcett, 2007), that this mechanism might be related to the impairment of the procedural memory network (Ullman and Pierpont, 2005).

Procedural memory is the implicit long-term memory system that stores the “blueprint” on how to perform learned motor and cognitive skills such as riding a bicycle (Ullman, 2001, 2004). The neural structures of the procedural memory network largely include: (1) the basal ganglia including the caudate and putamen; (2) the inferior frontal gyrus including the pars opercularis and pars triangularis; (3) the cerebellum; and (4) regions of the parietal cortex including the supramarginal gyrus (Ullman, 2004; Ullman and Pierpont, 2005; Mochizuki-Kawai, 2008; Doyon et al., 2009).

Procedural skills are a product of learning and consolidating sequence-based information (Ungerleider et al., 2002; Doyon, 2008; Doyon et al., 2009). According to Ullman, forming grammatical sentences utilizes these very processes. Specifically, Ullman argues that sentence construction is governed by sequences of morphosyntactic and phonological rules and conditions (Ullman, 2001, 2004). Following these rules in sequence ensures that the constructed sentence is grammatically coherent and thereby suggests that grammar is supported by the procedural memory network. Given this theoretical framework of the procedural model, Ullman and Pierpont predicted that procedural memory deficits might underlie the structural language deficits reported in several neurodevelopmental disorders including ASD and ADHD. This procedural deficit hypothesis (PDH) has been broken down by Ullman and Pierpont into two smaller testable hypotheses. One hypothesis proposes that differences in the neural structures underlying procedural memory are associated with differences in language ability. The other and most commonly tested hypothesis, argues that differences in language ability are associated with differences in learning on procedural memory tasks (Ullman and Pierpont, 2005). In this study, we aimed to address the first of the two smaller testable hypotheses, which suggests that differences in the neural structures underlying procedural memory are associated with differences in language ability. To date, no studies have examined this hypothesis in ASD, ADHD, and TD concurrently.

### Neural Structure and Language Associations in ASD and ADHD

There are a handful of studies that have examined associations between neural structures and language in ASD, some of which have reported significant associations with structures linked to procedural memory (e.g., De Fossé et al., 2004; Knaus et al., 2009; Hodge et al., 2010; Joseph et al., 2014). Specifically, studies have reported associations between the greater right volumetric asymmetry of the pars triangularis (Knaus et al., 2009), pars opercularis (Joseph et al., 2014), the inferior frontal gyrus, and supramarginal gyrus (De Fossé et al., 2004) and lower language scores in ASD. In contrast, one study reported an association between greater left volumetric asymmetry of lobule VIIIA of the cerebellum and lower language scores in ASD (Hodge et al., 2010). Other studies, however, have not found associations with these same structures, namely between the volumetric asymmetry of the pars triangularis (Joseph et al., 2014), pars opercularis (Knaus et al., 2009), cerebellum (Webb et al., 2009), and language scores in ASD.

To our knowledge, only one study has explored associations between neural structures and language in ADHD (Kibby et al., 2009). This study only examined the pars triangularis and found an extra sulcus in the pars triangulari of the children in the ADHD group, which was associated with lower language scores. With so few studies exploring neural structure and language associations in ASD and ADHD and no studies investigating these relationships in the context of the PDH, it remains unclear whether differences in the neural structures underlying procedural memory are associated with differences in structural language ability in ASD and ADHD.

### Study Objectives

To address this gap in the literature, we aimed to:

(1) Determine whether receptive and expressive structural language abilities differed between children with ASD, ADHD, and TD.
(2) Determine whether the thickness, area, or volume of the neural structures underlying the procedural memory network differed between children with ASD, ADHD, and TD using a region-of-interest analytic approach.
(3) Determine whether thickness, area, or volume of the neural structures underlying the procedural memory network were associated with the structural language abilities of children with ASD, ADHD, and TD using a whole-brain vertex and voxel-wise analytic approach.

## Materials and Methods

### Participants

One hundred and sixty-three children (ages 10–21 years) participated in this study: (1) 91 with ASD; (2) 26 with ADHD; and (3) 46 with TD. Participants were recruited from the Province of Ontario Neurodevelopmental Disorders (POND) Network, a multicenter research program aimed at understanding neurodevelopmental disorders including ASD and ADHD to facilitate the development of new and effective treatments (Baribeau et al., 2017). Participants with ASD were recruited and tested at Holland Bloorview Kids Rehabilitation Hospital in Toronto, the Hospital for Sick Children in Toronto, and Lawson Health Research Institute in London, Ontario. Participants with ADHD were recruited and tested at the Hospital for Sick Children in Toronto and participants with TD were recruited externally and tested at Holland Bloorview Kids Rehabilitation Hospital in Toronto and Queen’s University in Kingston. Informed consent was obtained for experimentation with human subjects from research ethics committees at all sites.

All participants met the following study inclusionary criteria: (1) had normal hearing; (2) scored at or above 70 on a non-verbal measure of intelligence; and (3) had a gestational age of 35 weeks or more.

To confirm clinical diagnoses, a series of standardized measures were administered to participating children and their parents. To verify the ASD diagnosis, the Autism Diagnostic Observation Schedule (ADOS; Lord et al., 2000) or ADOS-II (Lord et al., 2012), a diagnostic assessment of social, play/imagination, and communication skills, as well as restricted/repetitive interests/behaviors, was administered to the children with ASD and the Autism Diagnostic Interview-Revised (ADI-R; Lord et al., 1994), a structured interview that provides a diagnostic algorithm for autism, was administered to their parents. To verify the ADHD diagnosis, the Parent Interview for Child Symptoms (PICS; Ickowicz et al., 2006), a semi-structured interview assessing disruptive behavior disorders, was administered to the parents of children with ADHD. Children with ADHD were further classified into three subtypes: inattentive, hyperactive, and combined. Of the 26 children with ADHD who were recruited, 10 children were classified as inattentive, two were classified as hyperactive and 14 were classified as combined. The degree of severity was determined using the PICS symptom count, where a greater number of symptoms indicates a greater degree of severity. Of 18 symptoms listed on the PICS, the average number of symptoms reported was 9.1 (3.8). Children were considered typically developing (TD) if they had no history of neurodevelopmental, psychiatric, or neurologic disorder and no first-degree family history of neurodevelopmental disorder.

### Cognitive Measure

Cognitive ability was measured using the full-scale standard score of one of the following standardized assessments of general cognitive functioning: the Wechsler Abbreviated Scale of Intelligence (WASI; Wechsler, 1999), the Wechsler Abbreviated Scale of Intelligence—Second Edition (WASI-II; Wechsler, 2011), the Wechsler Intelligence Scale for Children—Fourth Edition (WISC-IV; Wechsler, 2003) or the Wechsler Intelligence Scale for Children—Fifth Edition (WISC-V; Wechsler, 2014). However, given the likelihood for language impairment in a subset of children with ASD and ADHD, we chose to only include non-verbal IQ scores in our statistical analyses. Significant differences in the mean non-verbal IQ scores were found across groups. A Kruskal–Wallis non-parametric ANOVA *post hoc* test revealed that the ASD and ADHD groups were not significantly different from one another, but were both significantly different from the TD group (Table 1).

**Table 1:** Behavioral characteristics across diagnostic groups.

	Group				
Characteristic	ASD (*N* = 92)	ADHD (*N* = 26)	TD (*N* = 47)	*p*	*Post hoc* comparisons
Age (years)
Mean (SD)	12.9 (2.7)	11.8 (1.5)	14.8 (3.7)	<0.01	ASD < TD
Range	10–21	10–16	10–21		ADHD < TD
Nonverbal IQ
Mean (SD)	105.1 (17.8)	103.48 (15.1)	113.5 (15.9)	0.013	ASD < TD
Range	71–150	70–124	77–154		ADHD < TD
Sex (male)
*N* (%)	69 (75.8)	19 (73.0)	26 (56.5)	<0.01	ASD > TD
					ADHD > TD

### Language Measure

Language ability was measured using the receptive and expressive language composite scores from the Oral and Written Language Scales—Second Edition (OWLS-II; Carrow-Woolfolk, 2011), the Clinical Evaluation of Language Fundamentals—Fourth Edition (CELF-IV; Semel et al., 2003) and, the Clinical Evaluation of Language Fundamentals—Fifth Edition (CELF-V; Wiig et al., 2013), standardized assessments of general language ability that assess morphology, syntax, and phonology (structural language) in receptive and expressive domains.

### MRI Acquisition and Processing

All participants were scanned at the Hospital for Sick Children in Toronto, Canada on a Siemens 3T scanner. During the study, the scanner underwent an upgrade from Trio Tim to Prisma. We covaried for the scanner to account for differences between sequences. T1-weighted MPRAGE scans were acquired with grappa acceleration. The scans from the Trio Tim had a TE of 2.96 ms, TR of 2,300 ms, and 1 mm^3^ isotropic voxels; where scans from the Prisma had a TE of 3.14 ms, TR of 1,870 ms, and 0.8 mm^3^ isotropic voxels.

Scans were processed with CIVET version 2.1 on CBRAIN (Ad-Dab’bagh et al., 2006; Sherif et al., 2014), after denoising with adaptive non-local means filtering (Manjón et al., 2010). CIVET comprises intensity normalization, n4 bias field correction, linear and nonlinear registration to the ICBM2009s symmetric non-linear template, gray/white matter surface extraction, surface registration, and cortical morphometry calculations.

CIVET scans were quality controlled in a semi-automatic fashion, using the CIVET quality control pipeline. The stringency of the quality control pipeline was marginally reduced to include scans with fewer than 150 surface-surface intersections and self-intersections per hemisphere. The adjusted threshold was determined based on an acceptable trade-off between the inclusion of high-quality scans and exclusion of low-quality scans identified by hand in a subset of the data. Also, scans, where at least one atlas structure was not identified in the segmentation, were not included in the analysis.

Thickness, area, and volume estimates for cortical structures of interest were extracted using the Automated Anatomical Labeling (AAL) atlas (Tzourio-Mazoyer et al., 2002). Scans were parcellated with Multiple Automatically Generated Templates Brain Segmentation Algorithm (MAGeT; Chakravarty et al., 2013) using the CoBrALab atlas suite (Chakravarty et al., 2006; Winterburn et al., 2013; Park et al., 2014; Tullo et al., 2018) for subcortical structures.

### Statistical Analysis

To compare structural language abilities across children with ASD, ADHD, and TD, regression analyses were conducted to determine whether receptive and expressive structural language scores differed across groups. We entered age into the model to control for the wide age range of participants, sex to account for group differences in sex distribution, and non-verbal IQ to control for group differences in non-verbal cognitive functioning and diagnosis. Analyses for receptive and expressive structural language scores were conducted separately. We applied the Benjamini-Hochberg correction (Benjamini and Hochberg, 1995) to control for the expected false discovery rate (FDR) at 0.05.

To compare neural structures across children with ASD, ADHD, and TD, regression analyses were conducted to determine whether diagnosis predicted differences in thickness, area, or volume of the neural structures supporting procedural memory. We entered age, sex, scanner to control for the differences between the two scanners that were used, whole-brain volume to control for non-specific differences in overall brain size across participants, non-verbal IQ, and diagnosis. The cortical and subcortical structures of interest included: (1) the inferior frontal gyrus; (2) the parietal gyrus; (3) the caudate; (4) the supramarginal gyrus; and (5) the subcomponents of cerebellum. Although the putamen is part of the procedural network, it is largely involved in motor learning (Ullman, 2001, 2004; Doyon et al., 2009). Given that we aimed to identify the role procedural memory plays in structural language, we chose to exclusively examine structures that are involved in cognitive procedural learning. Analyses for each structure were conducted separately and subject to FDR correction.

To examine if cortical anatomy was associated with receptive and expressive structural language ability, we conducted a whole-cortex vertex-wise analysis. At each vertex in the cortical mesh identified by CIVET the following analysis was conducted for the area, volume, and thickness separately. First, scanner was regressed out of the vertex measure. Then, receptive and expressive language scores were separately regressed against residual thickness, area, or volume with diagnosis, sex, age, and whole-brain volume as covariates. *P*-values for the effect of diagnosis were computed for each vertex and measures were computed and subject to FDR correction.

To examine if subcortical anatomy was associated with receptive and expressive structural language ability, we conducted a two-step regression analysis. These structures included the caudate and the subcomponents of the cerebellum. We first regressed scanner against subcortical structure volumes to obtain residual volumes. These residual volumes, as well as age, sex, whole brain volume, non-verbal IQ, and diagnosis, were entered as predictors into a second regression for receptive and expressive language scores. *P*-values were subject to FDR correction.

## Results

The regression analyses revealed that diagnosis uniquely predicted receptive, *F*_(1,161)_ = 11.00, *p* < 0.01, and expressive language scores, *F*_(1,161)_ = 10.53, *p* < 0.01, independent of age, sex and non-verbal IQ. Kruskal–Wallis *post hoc* comparisons revealed that the mean receptive language scores for the ASD and ADHD groups were not significantly different from one another, but were both significantly different from the TD group. Also, the mean expressive language scores for the ADHD and TD groups were not significantly different from one another but were both significantly different from the ASD group (Table 2).

**Table 2:** Receptive and expressive language abilities across diagnostic groups.

	Group				
Characteristic	ASD (*N* = 92)	ADHD (*N* = 26)	TD (*N* = 47)	*p*	*Post hoc* comparisons
Receptive language
Mean (SD)	94.1 (19.3)	95.65 (15.7)	108.1 (11.8)	<0.01	ASD < TD
Range	40–132	59–122	80–129		ADHD < TD
Expressive language
Mean (SD)	90.4 (20.5)	101.8 (13.7)	107.2 (12.4)	<0.01	ASD < ADHD
Range	40–133	73–130	79–133		ASD < TD

The regression analyses revealed that after FDR correction, diagnosis was not a significant predictor of the thickness, area, or volume of the structures comprising the procedural memory network (Table 3).

**Table 3:** Thickness (mm), area (mm^2^), and volume (mm^3^) of structures of interest by diagnostic groups.

		Group		
Measure	Structure of interest	ASD	ADHD	TD
		Mean (SD)	Mean (SD)	Mean (SD)
Thickness	Inferior frontal gyrus	(*N* = 91)	(*N* = 26)	(*N* = 45)
	Left opercularis	3.7 (0.3)	3.7 (0.1)	3.6 (0.3)
	Left traingularis	3.6 (0.3)	3.7 (0.1)	3.6 (0.3)
	Right opercularis	3.7 (0.2)	3.7 (0.2)	3.7 (0.2)
	Right triangularis	3.7 (0.2)	3.7 (0.2)	3.7 (0.2)
	Supramarginal gyrus	(*N* = 91)	(*N* = 26)	(*N* = 45)
	Left	3.7 (0.3)	3.7 (0.1)	3.5 (0.3)
	Right	3.7 (0.2)	3.6 (0.1)	3.6 (0.2)
Area	Inferior frontal gyrus	(*N* = 91)	(*N* = 26)	(*N* = 45)
	Left	3,570.2 (512.8)	3,569.8 (482.1)	3,508.2 (428.3)
	Right	3,449.4 (431.4)	3,589.5 (600.6)	3,511.8 (485.1)
	Supramarginal gyrus	(*N* = 91)	(*N* = 26)	(*N* = 45)
	Left	3,364.7 (528.7)	3,270.5 (439.4)	3,177.5 (470.2)
	Right	3,070.6 (504.8)	3,159.1 (450.1)	3,016.3 (493.0)
Volume	Whole brain	(*N* = 91)	(*N* = 26)	(*N* = 46)
		1,482.1 (147.2)	1,434.7 (136.8)	1,464.8(135.5)
	White matter	(*N* = 91)	(*N* = 26)	(*N* = 46)
		462.0 (64.2)	444.5 (60.7)	480.2 (52.4)
	Cortical grey matter	(*N* = 91)	(*N* = 26)	(*N* = 46)
		642.6 (68.1)	630.3 (63.1)	630.5 (71.4)
	Subcortical grey matter	(*N* = 91)	(*N* = 26)	(*N* = 46)
		102.7 (17.3)	100.0 (18.4)	88.3 (16.9)
	Caudate	(*N* = 90)	(*N* = 26)	(*N* = 43)
	Left	3,708.1 (500.3)	3,575.2 (576.4)	3,703.4 (434.5)
	Right	3,731.5 (498.4)	3,616.8 (584.0)	3,719.4 (468.7)
	Inferior frontal gyrus	(*N* = 87)	(*N* = 22)	(*N* = 40)
	Left	11,579.6 (1614.8)	11,398.9 (1613.5)	11,257.2 (1631.5)
	Right	11,177.6 (1517.4)	11,500.1 (2085.5)	11,281.3 (1834.6)
	Cerebellum
	Left	(*N* = 90)	(*N* = 25)	(*N* = 43)
	I, II	202.4 (58.2)	203.6 (49.3)	213.6 (59.3)
	III	557.4 (103.3)	541.4 (63.2))	563.9 (88.0)
	IV	1,784.9 (343.2)	1,749.0 (260.2)	1,862.7 (311.1)
	V	3,864.1 (575.2))	3,731.6 (420.4)	3,974.3 (412.1)
	VI	8,151.3 (1198.5)	7,898.8 (784.4)	8,254.6 (1127.0)
	Crus I	13,685.1 (1663.4)	13,422.0 (1660.9)	13,363.5 (1814.5)
	Crus II	9,352.3 (1344.6)	9,255.7 (1214.4)	9,371.9 (1572.2)
	VIIB	4,272.3 (737.4)	4,115.3 (486.6)	4,114.7 (487.6)
	VIIIA	5,593.0 (1015.5)	5,348.1 (661.4)	5,680.2 (779.0)
	VIIIB	3,544.4 (605.7)	3,450.5 (467.3)	3,603.6 (497.7)
	IX	3,578.2 (687.8)	3,566.9 (714.1)	3,481.4 (541.7)
	X	518.0 (58.2)	493.6 (61.4)	517.5 (62.1)
	Right
	I, II	202.4 (58.2)	203.62 (49.3)	213.6 (59.3)
	III	661.7 (124.0)	643.0 (76.8)	665.6 (118.2)
	IV	1,381.3 (261.3)	1,361.2 (210.3)	1,455.8 (240.4)
	V	3,944.1 (611.3)	3,794.8 (474.6)	4,125.8 (522.3)
	VI	8,640.0 (1243.6)	8,564.2 (790.5)	8,701.8 (1177.9)
	Crus I	13,748.4 (1769.6)	13,497.0 (1588.5)	13,630.5 (2078.7)
	Crus II	10,148.8 (1444.0)	9,815.2 (1055.3)	9,813.5 (1281.1)
	VIIB	5,149.1 (810.5)	4,961.2 (670.9)	5,036.6 (646.5)
	VIIIA	4,586.0 (815.7)	4,406.0 (506.1)	4,539.4 (550.9)
	VIIIB	3,462.1 (557.0)	3,379.5 (441.2)	3,517.5 (466.4)
	IX	3,451.1 (632.6)	3,372.5 (571.0)	3,405.4 (479.5)
	X	550.8 (75.7)	526.4 (51.9)	547.5 (86.0)
	Supramarginal gyrus	(*N* = 87)	(*N* = 22)	(*N* = 40)
	Left	10,718.0 (1775.7)	10,038.5 (1266.8)	9,870.9 (1570.8)
	Right	9,734.0 (1607.6)	9,755.5 (1395.1)	9,300.0 (1608.4)

**Figure 1:**
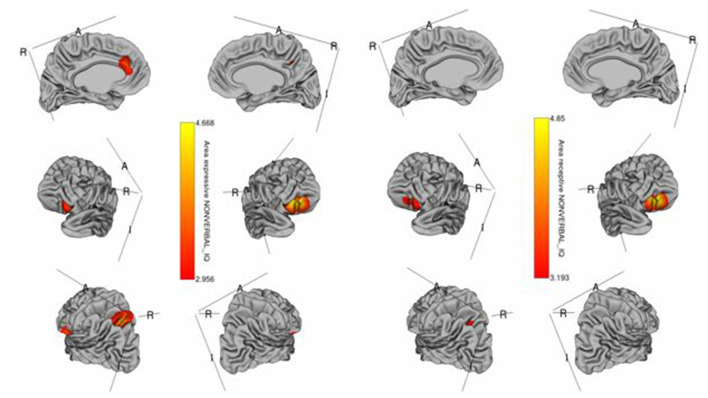
Brain regions where the cortical area is significantly associated with receptive (right) and expressive (left) language scores across diagnostic groups. Colored vertices depict *t*-statistics for the effect of the cortical area in vertex the specific regression where the vertex was significant after false discovery rate (FDR) correction.

**Figure 2:**
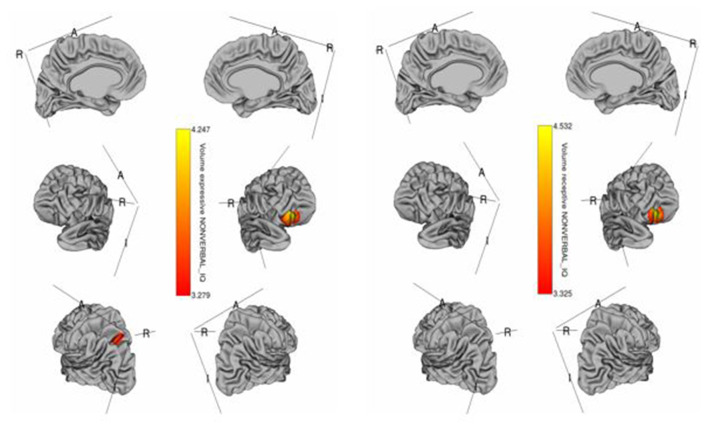
Brain regions where cortical volume is significantly associated with receptive (right) and expressive (left) language scores across diagnostic groups. Colored vertices depict *t*-statistics for the effect of cortical volume in vertex the specific regression where the vertex was significant after FDR correction.

The whole-cortex vertex-wise analyses revealed associations between several cortical structures and receptive and expressive language scores independent of age, sex, whole brain area/volume, non-verbal IQ, and diagnosis (Table 4). Notably, the area and volume of the right superior frontal gyrus, right inferior frontal gyrus, and right middle frontal gyrus were significantly associated with both receptive and expressive language scores across ASD, ADHD, and TD groups (Figures 1, 2). The area of the left superior parietal gyrus, left inferior frontal gyrus, left superior frontal gyrus, left anterior cingulate and paracingulate gyri, left angular gyrus and the left middle and superior occipital gyri were only associated with expressive language scores across ASD, ADHD, and TD groups (Figure 1).

**Table 4:** Area (mm^2^) and volume (mm^3^) of structures with significant vertices that predict expressive and receptive language abilities across diagnostic groups.

Measure	Language	Structure	Number of significant vertices
Area	Receptive	Right superior frontal gyrus	311
		Right inferior frontal gyrus	265
		Right middle frontal gyrus	98
	Expressive	Left superior parietal gyrus	615
		Right inferior frontal gyrus	345
		Left inferior frontal gyrus	326
		Right superior frontal gyrus	260
		Left anterior cingulate and paracingulate gyri	245
		Left angular gyrus	129
		Right middle frontal gyrus	108
		Left superior frontal gyrus	73
		Left middle occipital gyrus	14
		Left superior occipital gyrus	6
Volume	Receptive	Right inferior frontal gyrus	311
		Right superior frontal gyrus	154
		Right middle frontal gyrus	48
	Expressive	Right inferior frontal gyrus	208
		Right superior frontal gyrus	196
		Right middle frontal gyrus	37

Further, regression analyses indicated that the caudate and subcomponents of the cerebellum, two of the most critical subcortical structures underlying the procedural memory network, were not associated with receptive or expressive language scores.

## Discussion

This study aimed to explore the brain-behavior associations outlined by the PDH (Ullman and Pierpont, 2005) in children with ASD, ADHD and TD, which suggests that differences in the neural structures underlying procedural memory contribute to the structural language impairments observed in children with neurodevelopmental disorders including ASD and ADHD. To test this hypothesis: we (1) compared receptive and expressive language abilities; (2) compared the thickness, area, and volume of the neural structures underlying the procedural memory network; and (3) attempted to establish whether these neural structures were associated with structural language ability across children with ASD, ADHD, and TD.

Based on the PDH, we expected that receptive and expressive language abilities would differ between children with ASD, ADHD, and TD. Our analyses revealed that children with ASD and ADHD were comparable in their receptive language abilities, but were both significantly different from children with TD, who showed stronger receptive language abilities. This finding is consistent with previous studies that have reported structural language difficulties in the receptive domain in both ASD and ADHD populations (Tager-Flusberg, 2006; Sciberras et al., 2014). For expressive language abilities, however, children with ADHD and TD were comparable to one another, but were both significantly different from children with ASD, who showed weaker expressive language abilities. This finding is consistent with ADHD studies that have suggested that children with ADHD tend to show fewer challenges with expressive language relative to receptive language (e.g., Tannock and Schachar, 1996).

Next, we expected that the thickness, area, and volume of the neural structures comprising the procedural memory network would differ between children with ASD, ADHD, and TD. Our analyses yielded statistically non-significant group differences across the neural structures that comprise the procedural memory system. In the literature, results have varied. While some studies have reported differences in the inferior frontal gyrus as well as the caudate and cerebellum in ASD and ADHD relative to TD (Castellanos et al., 2002; Hill et al., 2003; Hollander et al., 2005; Langen et al., 2007; Valera et al., 2007), others have not (Pineda et al., 2002; Hazlett et al., 2005; Sussman et al., 2015). These findings could suggest that differences in thickness, area and volume do not exist across children with ASD, ADHD, and TD. However, a second and more likely explanation is that if only some children with ASD and ADHD exhibit structural language difficulties, there may not have been a sufficient number of those children in our sample to show potential group differences in the neural structures underlying the procedural memory network. As a result, it is unclear whether differences in the structural properties (thickness, area, and volume) of the brain regions supporting the procedural memory system contribute to the structural language deficits observed across ASD and ADHD.

Lastly, we expected that the thickness, area, and/or volume of the brain structures underlying the procedural memory system would be associated with the structural language abilities of children with ASD, ADHD, and TD. Our analyses showed that several structures were associated with receptive and expressive language abilities across diagnostic groups. Four of these structures are linked to the procedural memory network: (1) the right frontal gyrus—superior, middle, and inferior; (2) the left superior and inferior frontal gyrus; and (3) the left superior parietal gyrus. These structures have well-established roles in language comprehension and production. The right inferior frontal gyrus is known to be involved in language processing, broadly speaking (Doyon et al., 1997; Conway and Christiansen, 2001; Ullman, 2004) and is, thus, consistent with our results that indicate that the area and volume of this structure predict both receptive and expressive language abilities. Its left homolog, however, the left inferior frontal gyrus, plays a more active role in the articulatory network, processing phonological information, which would explain why it surfaced as an exclusive predictor of expressive language ability (Holland et al., 2007; Kadis et al., 2008; Pang et al., 2011). The left superior parietal gyrus and the left superior frontal gyrus have both been linked to working memory (Boisgueheneuc et al., 2006; Koenigs et al., 2009). Working memory houses the phonological loop, which is said to process and temporarily store articulatory information (Baddeley and Hitch, 1974). It is, therefore, not surprising that these structures would also predict expressive language abilities.

The remaining structures that our analyses revealed were associated with language abilities are not known to be part of the procedural memory network. These structures include: (1) the left angular gyrus; (2) the left superior and middle occipital gyri; and (3) the left anterior cingulate and paracingulate gyri. Some of these structures have been linked to language phenomena, namely the left angular gyrus, the left superior and middle occipital gyri. The left angular gyrus is commonly associated with semantic processing of oral speech (Binder et al., 2009) and is linked to the declarative memory network. Language abilities that are not driven by rules or sequences, such as lexical-semantic knowledge, are arguably supported by the declarative memory system, an explicit long-term memory system that forms, stores, and retrieves fact-based information. This memory system is linked to lexico-semantic development including vocabulary acquisition (Ullman, 2001, 2004; Ullman and Pierpont, 2005). Given that some vocabulary is incorporated within the listening comprehension and oral expression scales of the OWLS-II, it is not surprising that we would find structures associated with declarative memory also surface as predictors of language abilities. Some studies have speculated that the middle and superior occipital gyri are involved in identifying letters and character sequences in word reading as well as transferring that information to the left posterior superior temporal gyrus (Wernicke’s area) for processing (Sakurai, 2004; Levy et al., 2009). Furthermore, research has shown reduced activity in at least the middle occipital gyrus in Dyslexia, a learning disorder that primarily impairs the ability to read and write (Paulesu et al., 2001).

The structures that are not typically related to language processing, but were found to be associated with structural language abilities include the areas of the anterior cingulate and paracingulate gyri as well as the right superior and middle frontal gyri. These structures have been linked to different forms of executive function. Specifically, the anterior cingulate and paracingulate gyri have been reported to be involved in conflict resolution and resource allocation (Abutalebi et al., 2013; Gennari et al., 2018). The right superior and middle frontal gyri, on the other hand, have been reportedly involved with reorienting attention (Japee et al., 2015) and response inhibition (Aron et al., 2003). There is growing evidence that executive functions contribute to language processing (Kaushanskaya et al., 2017) and is, therefore, not unusual that these structures would be engaged while completing language tasks. It is, however, unclear why these structures were associated with expressive language abilities only and not associated with receptive language abilities.

Our findings show that in the presence of group differences in structural language, the neural structures that make up the procedural memory network were not structurally different across ASD, ADHD, and TD groups. Further, very few structures that comprise the procedural memory network were associated with structural language ability and those that did, like the inferior frontal gyrus, have well-established roles in language processing and production (Ardila et al., 2015) independent of their role in procedural learning and as part of the procedural memory system. Subcortical structures such as the caudate of the basal ganglia and cerebellum, which arguably play critical roles in procedural learning, were not associated with structural language abilities across diagnostic groups. Based on these findings, it is unclear whether procedural memory plays a fundamental role in structural language learning in ASD, ADHD, and TD.

### Limitations

Though these findings contribute to our understanding of the procedural memory network in ASD and ADHD, there are some limitations to consider. First, we did not use a measure of procedural memory. The purpose of this study was to test the PDH-derived hypothesis that differences in the neural structures underlying procedural memory are related to differences in structural language abilities. Generally, procedural memory studies aim to make inferences about the state and function of the neural correlates supporting procedural memory based on performance on procedural memory tasks such as the serial reaction time task. Here, we were able to make conclusions about the structural parameters of these neural correlates because we examined these structures directly.

Second, we did not consider the entire range of structural language ability across ASD and ADHD. One of the study’s inclusionary criteria was that participants were required to have a non-verbal IQ standard score of 70 or above. This would have preemptively narrowed our subset of children with ASD and ADHD to children with mild to moderate structural language impairments and left the majority of children with severe structural language impairments out of our sample. Future studies need to evaluate the impact that intellectual disability has on the relationship between neural structures supporting procedural memory and structural language ability.

## Conclusions

This study is the first to test the procedural deficit hypothesis (Ullman and Pierpont, 2005) in ASD, ADHD and TD by comparing the structural language abilities, the neural structures underlying the procedural memory network, and the relationship between structural language and neural structure across diagnostic groups. We found differences in receptive and expressive structural language abilities but did not find differences in the neural structures underlying the procedural memory network in ASD, ADHD, or TD. Exploring the relationships between structural language abilities and neural structure revealed that the area and volumes of some structures supporting the procedural memory network, namely the inferior frontal gyrus and the superior parietal gyrus, were associated with individual differences in expressive and receptive structural language abilities across ASD, ADHD, and TD. These findings provide some support for the theoretical framework of the PDH, which suggests that structures supporting procedural memory underlie structural language learning. More importantly, however, our results inform our understanding of the neural basis of language by revealing other structures not traditionally associated with language learning in typical development and in ASD and ADHD.

## Data Availability Statement

The raw data supporting the conclusions of this article will be made available by the authors, without undue reservation.

## Ethics Statement

The studies involving human participants were reviewed and approved by the Research Ethics Boards at all sites. Written informed consent to participate in this study was provided by the participants’ legal guardian/next of kin.

## Author Contributions

TS: conceptualization and writing. CH: analysis and writing. JB, JC, RS, EK, XL, RN, and JL: conceptualization, writing, and reviewing. AI: data curation, writing, and reviewing, SD and LR: implementation, writing, and reviewing. EA: supervision, conceptualization, writing, and reviewing. All authors contributed to the article and approved the submitted version.

## Conflict of Interest

EA has received consultation fees from Roche and Quadrant, research funding from Roche, in-kind supports from AMO pharma, editorial honoria from Wiley and book royalties from APPI and Springer. She holds a patent for the device, “Tully” (formerly Anxiety Meter). She has received royalties from APPI and Springer. RS has consulted to Highland Therapeutics, Eli Lilly and Co., and Purdue Pharma. He has commercial interest in a cognitive rehabilitation software company, “eHave”. RN has received research grants from Roche.
